# Psychometric Properties of the Brief COPE Inventory on a Student Sample

**DOI:** 10.3390/bs15111579

**Published:** 2025-11-18

**Authors:** Julia A. Marakshina, Sofia A. Mironets, Anna A. Pavlova, Victoria I. Ismatullina, Marina M. Lobaskova, Anna A. Pecherkina, Elvira E. Symaniuk, Sergey B. Malykh

**Affiliations:** 1 Laboratory of Population Research, Ural Federal University, 620062 Yekaterinburg, Russia; 2 Neurocognitive Laboratory, Dmitry Rogachev National Medical Research Center of Pediatric Hematology, Oncology and Immunology, 117198 Moscow, Russia; 3 Developmental Behavioral Genetics Laboratory, Federal Research Centre of Psychological and Interdisciplinary Studies, 125009 Moscow, Russia

**Keywords:** psychometric properties, coping strategies, students, COPE

## Abstract

The aim of this study was to evaluate the psychometric properties of the Brief Coping Orientation to Problems Experienced (COPE) inventory among students. The data was collected via an online platform from Russian universities involving first–fourth-year students (N = 670). The participants completed the Brief COPE inventory (32 items). Of these, 529 (79%) were female, and 141 (21%) were male. The age range was 18 to 29 years. For this study, the inventory was modified, and its reliability (internal consistency) and validity (internal, external) were assessed. Participants were asked also to complete three additional tests: the Perceived Stress Scale (PSS), the Hospital Anxiety and Depression Scale (HADS), and the Mental Toughness Questionnaire (MTQ-10). Principal Component Analysis (PCA) and Confirmatory Factor Analysis (CFA) showed that the data fit the expected six-factor structure after a reduction of seven items with low factor loadings from the inventory structure. The internal consistency was good (Cronbach’s α in the range of 0.65–0.91). External validity demonstrated weak but significant indicators: all correlations between most scales of the brief COPE inventory and the scores of other tests ranged from 0.067 to 0.364). The Brief COPE, comprising 25 items, is a new tool for assessing the coping strategies of Russian students. It is a reliable, valid, accurate, and acceptable measure of coping strategies that can be used in large-scale studies in Russia. Additionally, the developed instrument may be potentially useful for application in educational and psychological screening, which opens up further opportunities for its practical implementation.

## 1. Introduction

Identifying stress coping strategies in students is important problem because students regularly experience various stressors—both universal ones, characteristic of the general population, and specific ones that affect only young people studying at universities (Zhdanov et al., 2020; Mironova, 2021; Kononov & Novikova, 2023). Universal stressors include economic crises, the death of a loved one, the need to start a family, illnesses, moving, etc. Exposure to specific stressors is conditioned by the very situation of studying at a university: academic loads, including extreme ones related to overload and the periodic need to take exams and tests, the necessity of building relationships with peers and teachers, which, due to the nature of university education, change quite frequently. Furthermore, the nature of stressors can change throughout the entire period of study. In the first year, stress can be caused by the situation of adapting to a new type of education, different from school. In the middle of their studies, students may encounter the so-called “third-year crisis,” when the need for further self-determination becomes acute due to specialization. Final-year students experience stress due to the need to complete and defend their graduation thesis, as well as due to the uncertainty associated with the moment of graduation and the need to find a job.

Choosing an effective coping strategy can significantly reduce the negative consequences of students’ stress load and preserve their mental health (Evans-Lacko et al., 2018; Kecojevic et al., 2020). Studying an individual’s coping strategies and optimizing their application in accordance with a person’s individual psychological characteristics is an important aspect of preventive and psychotherapeutic work in the student population (Knoll et al., 2005; Karyotaki et al., 2020). A relevant scientific problem is the choice of a high-quality diagnostic tool that determines the structure of effective and ineffective coping strategies in individuals of student age.

Lazarus and Folkman (Lazarus & Folkman, 1984) distinguish between two primary coping approaches: problem-focused strategies, which target the external source of stress in order to remove or reduce it, and emotion-focused strategies, which center on managing emotional responses. Yet, more recent perspectives suggest that limiting coping styles to just these two categories oversimplifies the complexity of coping behaviors (Carver et al., 1989). In response to this limitation, Parker and Endler (Endler & Parker, 1990) proposed a three-factor model consisting of task-oriented coping (efforts to resolve the problem or mitigate its impact, including cognitive reframing), emotion-oriented coping (heightened emotional reactions, self-focused attention, and fantasy-based escape), and avoidance-oriented coping (distraction and engagement in alternative activities).

The need to identify stress coping strategies in students necessitates the development of special questionnaires aimed at meeting this goal. Among such tools, the COPE inventory (Carver et al., 1989) and its brief version (Carver, 1997) can be highlighted. The COPE inventory is designed to measure both situational coping strategies and the underlying dispositional styles. A literature review shows that this inventory is widely used in different population groups and in various countries (Guan et al., 2020; Graves et al., 2021; Aragonès et al., 2023; Rijal et al., 2023). It is worth mentioning separately that the analysis of the psychometric properties of the inventory has been applied to student samples in various countries, including the short version (Brief COPE): the USA, China, Spain, and others (Miyazaki et al., 2008; Cheng et al., 2025; Fernández-Martín et al., 2022; Pels et al., 2023; Alotaibi et al., 2024; Solberg et al., 2024; Yusoff, 2010).

The full adaptation of the Russian-language version of the COPE inventory was presented in two variants: by Ivanov and Garanyan (2010) and by Rasskazova et al. (2013) (Ivanov & Garanyan, 2010; Rasskazova et al., 2013). The present study used the second version, as it demonstrated higher reliability indicators (Cronbach’s α coefficient). Based on it, the validation of a brief modification was carried out. Our task was to develop a brief version of the COPE inventory and test its psychometric properties. The advantages of a brief version are that it can be used in a battery of tests alongside other methods, as the short version requires less time to complete. This is convenient for both researchers and practicing psychologists, as well as education professionals. In this case, it is possible to obtain data not only on the nature of the coping strategies used by students but also on other psychological characteristics, which can be useful for both theoretical research and practical diagnostics.

Thus, the aim of the study was to modify the Coping Orientation to Problems Experienced (COPE) inventory, namely, to develop a brief version and test its psychometric properties (reliability, factor structure, external validity) on a student sample. Drawing on prior research, both domestic and international, which shows variation in the identified factor structures (Solberg et al., 2021), we intend to adapt a shortened six-factor version of the questionnaire, informed by the results of international studies and our own empirical findings (Pavlova et al., 2022; J. Marakshina et al., 2023).

## 2. Materials and Methods

### 2.1. Sample

Data was collected via an online platform in Russia. Consent was obtained from the universities’ administration teams to conduct testing on university grounds. Some students were tested at home on an individual basis or during an online lesson under the control of coordinators. Data collection was carried out on personal computers. These participants were part of the study investigating the individual differences in student psychological stress. The initial data pool included responses from 1202 respondents. Subsequently, an analysis was conducted to remove the responses of students who did not complete all questionnaires, left items blank, provided incomplete answers, or responded too quickly. The final dataset comprised 670 observations. Of these, 529 (79%) of the participants were female, and 141 (21%) were male. The age range was 18 to 29 years (mean age 19.89, standard deviation 2.21, median 19.0). A total of 267 (40%) students were in their first year, 154 (23%) in their second year, 146 (22%) in their third year, 80 (12%) in their fourth year, 14 (2%) in their fifth year, and 9 (0.01%) in their sixth year. In terms of academic programs, 387 (58%) students were enrolled in a bachelor’s program, 245 (37%) in a specialist program, 37 (6%) in a master’s program, and 1 student was a postgraduate (PhD) student. The study participants received their first higher education. Students studied STEM disciplines (7%), humanities (61%), and life science (32%).

All respondents provided informed consent to participate in the study. The study was approved by the Ethics Committee of the Ural Federal University named after the first President of Russia B.N. Yeltsin (Protocol No. 4, approval date 20 September 2023).

### 2.2. Measures

*Coping Orientation to Problems Experienced (COPE) Inventory.* The study used a brief version of the inventory, developed based on the Russian adaptation of Carver’s methodology (Rasskazova et al., 2013) performed by Rasskazova et al. In the first stage, highly correlated scales were merged, resulting in six general scales: “Mental disengagement”, “Active coping” (which included the classic COPE scales: Active coping, Planning, Denial, Behavioral Disengagement and Suppression of competing activities), “Socio-Emotional Support” (combining the scales: Seeking social support for emotional reasons, Seeking social support for instrumental reasons, Focus on and venting of emotions), “Turning to Religion”, “Positive Coping” (Positive Reframing and Growth, and Humor), and “Acceptance” (Appendix A Table A1). The correlation values ranged from 0.22 to 0.77 in absolute value (for details see Rasskazova et al., 2013). Items from the “Distraction” and “Denial” scales were inverted due to their contradictory meaning within the “Problem-focused coping” scale. An item, “I use alcohol or drugs to think less about it,” was also added to measure the “Substance Use” strategy, as in Carver’s original version (Carver et al., 1989). In the second stage, items with low factor loadings were removed from each scale. Most of the factor loadings ranged from 0.43 to 0.9 (for details see Rasskazova et al., 2013). The final scales consisted of 4–5 items each. Responses were assessed using a Likert scale with four categories: “Usually no, never,” “Rarely,” “Quite often,” and “Yes, often.” Each response option was assigned a quantitative equivalent ranging from 1 to 4 points. For items with reverse wording, a score reversal procedure was performed (1 = 4, 2 = 3, 3 = 2, 4 = 1). Composite scores for each scale were calculated as the arithmetic mean of the corresponding item scores.

*Perceived Stress Scale (PSS).* The 10-item Perceived Stress Scale (PSS) (Ababkov et al., 2016) was used to assess the level of perceived stress. This questionnaire, based on a 5-point Likert scale, measures the degree to which situations in life are appraised as stressful and overwhelming. The Russian adaptation includes two subscales: “Overstrain” (subjective intensity of daily stress) and “Stress management” (perceived helplessness in coping with difficulties). The total score reflects the overall level of stress, where higher scores on all scales indicate greater stress severity. In a previous study, the instrument demonstrated high reliability: Cronbach’s α was 0.86 for “Overstrain”, 0.78 for “Stress management”, and 0.88 for the total score (Pavlova et al., 2022).

*Mental Toughness Questionnaire (MTQ-10).* Mental toughness was assessed using the short 10-item Mental Toughness Questionnaire (MTQ-10) (Dagnall et al., 2019) with a 5-point Likert scale. This instrument has satisfactory psychometric properties and cross-cultural applicability. For a Russian adolescent sample, the reliability of the method was acceptable (Cronbach’s α = 0.73) (J. Marakshina et al., 2023).

*Hospital Anxiety and Depression Scale (HADS)* (Zigmond & Snaith, 1983). The Russian-language version was used (Y. A. Marakshina et al., 2025). The questionnaire consists of 14 items forming two subscales (7 items each): 1. Depression: assesses the severity of depressive symptoms (normal, subclinical, and clinical levels); 2. Anxiety: determines the level of anxiety symptoms (from normal to clinically significant). Responses are recorded on a 4-point Likert scale. A key feature of this instrument is its focus on assessing a patient’s emotional state regardless of somatic illness.

### 2.3. Statistical Analysis

The first stage of analysis involved the Principal Component Analysis (PCA) with Varimax rotation. Confirmatory Factor Analysis (CFA) was also conducted. The following fit indices were used to evaluate the model: Root Mean Square Error of Approximation (RMSEA), Comparative Fit Index (CFI), Tucker–Lewis Index (TLI), and Standardized Root Mean Square Residual (SRMR). The next step in the analysis was assessing internal consistency using Cronbach’s alpha. External validity was measured using Pearson correlation coefficients. Correlation coefficients were interpreted as: 0.90 to 1.00 (−0.90 to −1.00)—very high positive (negative) correlation; 0.70 to 0.90 (−0.70 to −0.90)—high positive (negative) correlation; 0.50 to 0.70 (−0.50 to −0.70)—moderate positive (negative) correlation; 0.30 to 0.50 (−0.30 to −0.50)—low positive (negative) correlation; 0.00 to 0.30 (0.00 to −0.30)—negligible correlation (Mukaka, 2012).

Descriptive statistics were calculated. Differences between genders were then assessed by comparing mean values. All statistical analyses were performed in R version 4.2.1 and JASP version 0.95.0.

## 3. Results

### 3.1. Principal Component Analysis (PCA)

**Figure 1:**
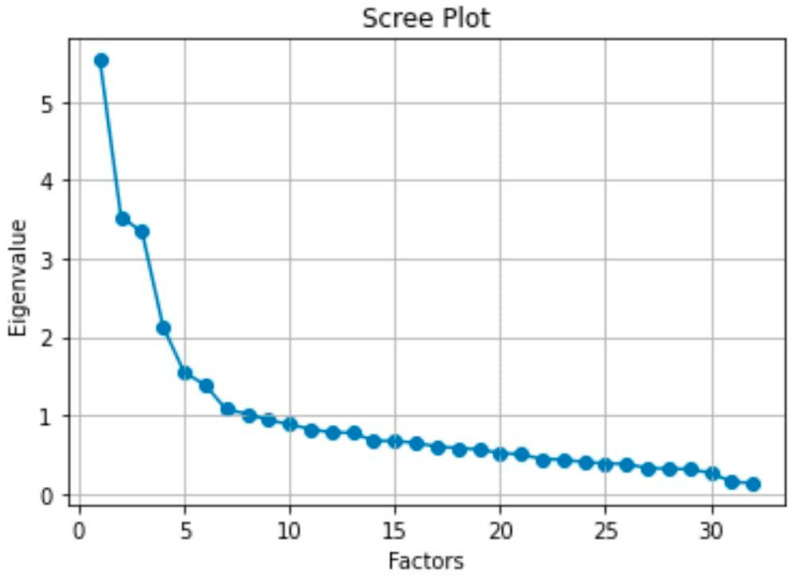
Scree plot for PCA. Six factors were determined.

Initially, we merged scales based on high correlations between them. A Principal Component Analysis (PCA) with Varimax rotation was conducted to analyze the inventory’s structure. The preliminary factor analysis revealed 6 factors (Figure 1), which together accounted for 56% of the variance.

During the model refinement process, items with low or incorrect factor loadings (questions 1, 5, 15, 18, and 20) were excluded, which improved the factor structure.

The following questions were thus removed:

“I turn to work or other substitute activities to take my mind off things.”

“I drink alcohol or take drugs, in order to think about it less.”

“I try to see it in a different light, to make it seem more positive.”

“I give up the attempt to get what I want.”

“I look for something good in what is happening.”

The final model identified the following factors (Table 1):

Turning to Religion—includes 4 items reflecting the use of religious practices and faith (loadings 0.86–0.88).

Socio-Emotional Support—combines 6 items related to seeking emotional and social support (loadings 0.54–0.75).

Acceptance—4 items describing acceptance of the situation (loadings 0.62–0.77).

Problem-focused coping—6 items related to taking active steps to overcome difficulties (loadings 0.40–0.69; one reverse-scored item showed a negative loading).

Avoidance—5 items reflecting the use of distracting strategies (loadings 0.34–0.64).

Humor—2 items concerning a humorous outlook on the situation (loadings 0.68 and 0.83).

Also, items 7R (“I reduce the amount of effort I’m putting into solving the problem”, Behavioral disengagement in Initial Subscales) and 3R (“I say to myself “this isn’t real”, Denial in Initial Subscales) were removed due to extremely low factor loadings (≤0.35). In addition, items 3 and 13 are similar in meaning, so it is possible to delete item No. 3. For instance, the item No. 22 “I go to movies or watch TV, to think about it less” had no analogs, so it was decided to leave it in the scale structure. Other items were retained based on theoretical reasoning. As a result, the original version was shortened to 25 items.

Thus, PCA confirmed a six-factor structure of coping strategies. Each factor has a meaningful interpretation and includes theoretically justified groups of items. The final model explains more than half of the variable variance, which can be considered a satisfactory indicator. It was decided to retain the 6-factor solution, as in the original questionnaire, which was subsequently tested using CFA.

### 3.2. Confirmatory Factor Analysis (CFA)

**Figure 2:**
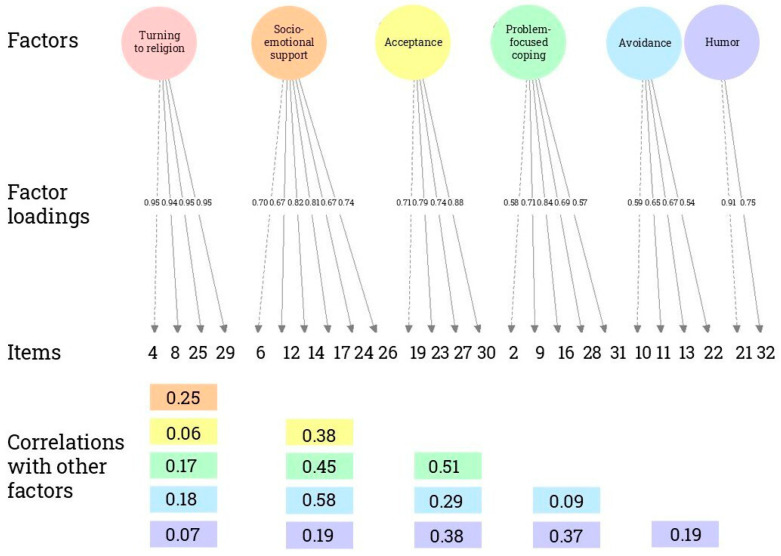
Path diagram (CFA model) for Brief Cope.

At the next stage, the 6-factor model was tested. Our modified questionnaire (Brief COPE), consisting of 25 items, includes 6 scales: “Avoidance” (4 items), “Problem-focused coping” (5 items), “Socio-Emotional Support” (6 items), “Turning to Religion” (4 items), “Humor” (2 items), and “Acceptance” (4 items) (Figure 2).

The following standard fit indices were used to assess the model fit: Standardized Root Mean Square Residual (SRMR) < 0.07, Tucker–Lewis Index (TLI) values close to 1, Comparative Fit Index (CFI) > 0.95, and Root Mean Square Error of Approximation (RMSEA) < 0.08 (Table 2).

The final CFA factor loadings for the 6-factor model are presented in Table 3. The Confirmatory Factor Analysis allowed for an assessment of the quality of the shortened questionnaire structure. The fit criteria for the original 6-factor model meet the established threshold values. Thus, the model demonstrates a good fit to the data and can be considered adequate and reliable (CFI = 0.988, TLI = 0.986; RMSEA = 0.057).

### 3.3. Internal Consistency

The Cronbach’s alpha reliability coefficients for all scales exceed 0.7, except for the “Avoidance” scale: “Turning to Religion”—0.91; “Socio-Emotional Support”—0.82; “Acceptance”—0.80; “Problem-focused coping”—0.76; “Humor”—0.73; “Avoidance”—0.65 (Table 3). This generally indicates high internal reliability of the questionnaire. The content of the “Avoidance” scale, whose reliability is borderline, requires clarification.

### 3.4. External Validity

In our study, the external validity of the short version of the Coping Strategies Questionnaire (Brief COPE) was assessed using Pearson correlations with the results from the following scales: the Perceived Stress Scale (PSS); the Hospital Anxiety and Depression Scale (HADS); and the Mental Toughness Questionnaire (MTQ-10).

As a result, significant correlations were obtained for the majority of the COPE scales (Table 4). Exceptions were the correlation values between the “Turning to Religion” scale and the HADS Depression scale, as well as the Perceived Stress Scale. Correlations between the “Socio-Emotional Support” scale and HADS Depression, the “Acceptance” scale and HADS Anxiety, as well as perceived stress, did not reach significance. Non-significant correlations were found between the “Avoidance” scale and mental toughness, and the “Humor” scale and HADS Anxiety. Significant correlations ranged from 0.067 to 0.364 (absolute values).

### 3.5. Descriptive Statistics: Gender Differences

The skewness and kurtosis of all scales fall within the range of −1 to +1, meaning all scales of the Brief COPE have a normal distribution. The next step in our analysis was to assess differences based on gender. The results are presented in Table 5. Overall, the mean scores for the “Socio-Emotional Support” (t = −4.68, *p* < 0.001), “Turning to Religion” (t = −3.31, *p* < 0.001), and “Avoidance” (t = −2.30, *p* < 0.02) scales were significantly higher in women compared to men (Table 6).

## 4. Discussion

The validation process identified a 6-factor structure for the Brief COPE inventory, consisting of 25 questions corresponding to the scales: “Avoidance” (4 items), “Problem-focused coping” (5 items), “Socio-Emotional Support” (6 items), “Turning to Religion” (4 items), “Humor” (2 items), and “Acceptance” (4 items) (Appendix A Table A2). Results of psychometric studies conducted in other countries demonstrate variations in the factor structure of the Brief COPE, which may be related to the sensitivity of the measured construct to cross-cultural differences, specifics of the validation procedure, and sample characteristics—such as size, gender composition, and other demographic indicators (Baumstarck et al., 2017; Wang et al., 2020; Solberg et al., 2024; Cheng et al., 2025). The methodologies for developing questionnaires vary considerably. As noted by Solberg et al. (2021), common practices include the a priori exclusion or modification of items/subscales prior to dimension reduction. An alternative method is scale reduction, where the original number of scales is maintained but condensed into two-item measures. This approach aims to preserve the measured constructs while reducing participant burden. However, measuring complex constructs like those in the COPE inventory with just two items may compromise validity. A single construct is best captured by assessing a variety of behaviors, and two-item scales are psychometrically problematic due to typically low reliability. This is supported by Yuan et al. (2017), who reported low reliability coefficients and unstable factor loadings for a two-item version of the Brief COPE, a concern echoed by Serrano et al. (2021). Consequently, the reliability of such simplified measurements may be overstated. The meta-analysis examines short versions adapted in various countries (Solberg et al., 2021). According to the author, the versions differ significantly in structure due to different approaches to shortening the questionnaire. The author also notes variations in cultural norms, which may also influence the number of factors affecting the interpretation of the wording. Our version of the inventory was developed based on a scale consolidation approach: correlated scales were merged into broader factors. We confirmed the theoretical structure comprising 6 coping strategies; however, not all questions demonstrated the ability to measure the intended constructs and were therefore removed. The following questions were excluded: “I turn to work or other substitute activities to take my mind off things” (Self-distraction), “I give up the attempt to get what I want”, “I say to myself “this isn’t real” (Active Coping); “I drink alcohol or take drugs, in order to think about it less” (Substance Use); “I try to see it in a different light, to make it seem more positive”, “I look for something good in what is happening” (Positive coping). The factor loadings of these items demonstrated values below the acceptable threshold of 0.35 and were thus excluded from the inventory. The insufficient factor loading for the question on Substance use may be because it is a unique item within the inventory structure, and it is not enough to form a full-fledged factor (it does not work “alone”). Furthermore, its diagnostic value might be distorted by students’ tendency to provide socially desirable responses, as the use of medication to address mental health issues, particularly emotional and personal problems, is still not very common in Russia. However, the removal of the item related to the Substance use scale, as well as the items of the Positive Reframing scale, which we theoretically included in the Humor scale, and other deleted questions, improved internal (in particular, factor) validity.

The Cronbach’s alpha coefficients for the inventory scales reach or exceed 0.7, demonstrating sufficient reliability, with the exception of the Avoidance scale, whose coefficient is 0.65 and may be interpreted as borderline acceptable reliability. It should be noted that the reliability of this scale, analyzed on a sample of teachers in a similar study of the Brief COPE’s psychometric properties, was also insufficient (and even lower) (Pavlova et al., 2022). Given the importance of this scale and the measurement of avoidance strategy, which is emphasized in other studies by its identification as a second-order scale, we consider it necessary to retain it within the inventory. A reliability coefficient approaching 0.7 allows for this.

The external validity of the Brief COPE was assessed using correlations with questionnaires measuring related constructs: PSS (level of perceived stress), HADS (indicators of anxiety and depression), and MTQ (mental toughness). The correlations demonstrated a small effect size; however, this is consistent with results obtained in other studies (Serrano et al., 2021). The direct nature of the correlations between some coping strategies and scales measuring emotional disorders (anxiety, depression), as well as the level of perceived stress, may indicate that individuals using these strategies are less effective at coping with stress. According to our analysis, these include: Turning to Religion, Socio-Emotional Support, Avoidance and Anxiety levels; Turning to Religion, Avoidance and Depression levels; Socio-Emotional Support, Avoidance and Perceived Stress levels. Conversely, the inverse correlations of Problem-focused coping with Anxiety, Depression, and Perceived stress; Acceptance with Depression; and Humor with Depression and Perceived Stress, suggest that students who use these coping strategies manage stress more effectively. Interestingly, the correlations between all Brief COPE scales and the MTQ questionnaire were positive except for the Avoidance scale which demonstrated no significant correlation with MTQ. This reflects that any coping strategy can be assessed from the standpoint of a resource that forms the potential for developing mental toughness. That is, the use of any coping strategy creates a foundation for building mental toughness.

A comparative analysis of the results for males and females shows that significant differences were found for the Avoidance, Socio-Emotional Support, Turning to Religion, and Humor scales. Females demonstrated higher scores on the Avoidance, Socio-Emotional Support, and Turning to Religion scales, while males scored higher on the Humor scale. Interestingly, the use of Avoidance, Socio-Emotional Support, and Turning to Religion, as shown above, have positive correlations with Anxiety, Depression, and Perceived Stress, meaning they are used by students who cope less effectively with stress. This is consistent with the results of earlier studies (Sadaghiani, 2013). Another view is that people who experience more stress and anxiety use more coping strategies. However, it is also possible that people with high levels of depression lack the resources to employ a variety of coping strategies. The results of a recent study support this: depressed patients tend to use avoidant coping strategies (e.g., Denial, Disengagement) more often, and proactive strategies (e.g., Active Coping, Planning) less often than healthy people, highlighting a significant behavioral difference between the groups (Orzechowska et al., 2013). The higher scores of females on Avoidance, Socio-Emotional Support, and Turning to Religion scales indicate a greater prevalence of these strategies in this group. This may also be related to the higher prevalence of anxiety and depressive disorders among women (Remes et al., 2016; Kuehner, 2017). In contrast, the Humor coping strategy is used more by males, has negative correlations with Depression and Perceived Stress, reflecting more effective stress coping in the male group, which creates a necessary buffer against the development of depressive disorders.

## 5. Limitations

This study has several limitations that should be considered when interpreting the results. Firstly, the sample is characterized by a pronounced gender imbalance, which may affect the generalizability of the findings and limit the ability to detect robust gender differences. In this regard, a promising direction for future research would be to create more balanced samples or to use statistical methods that can compensate for this disproportion. Secondly, the participants are representatives of only two cities. The results of the study would be enriched by including participants from other cities, both large (including the capital) and relatively small, which also have universities and their branches. Thirdly, the study did not test for measurement invariance, which limits the confidence in the comparability of the constructs across different groups, including gender. Future research prospects include the assessment of test–retest reliability.

## 6. Conclusions

The brief version of the Coping Orientation to Problems Experienced (Brief COPE) is a new tool for assessing the coping strategies of Russian students, has satisfactory psychometric properties, and may be recommended for practical use in theoretical and applied research.

## Figures and Tables

**Table 1:** Final Factor Loadings (PCA).

No.	Item	Factor 1. Turning to Religion	Factor 2. Socio- Emotional Support	Factor 3. Acceptance	Factor 4. Problem- Focused Coping	Factor 5. Avoidance	Factor 6. Humor
4	I seek God’s help.	0.87					
8	I put my trust in God.	0.86					
25	I try to find comfort in my religion.	0.88					
29	I pray more than usual.	0.87					
6	I try to get emotional support from friends or relatives.		0.62				
12	I let my feelings out.		0.54				
14	I try to get advice from someone about what to do.		0.74				
17	I get sympathy and understanding from someone.		0.75				
24	I get upset and let my emotions out.		0.55				
26	I talk to someone who could do something concrete about the problem.		0.68				
19	I get used to the idea that it happened.			0.62			
23	I accept that this has happened and that it can’t be changed.			0.76			
27	I learn to live with it.			0.62			
30	I accept the reality of the fact that it happened.			0.77			
2	I concentrate my efforts on doing something about it.				0.59		
7R	I reduce the amount of effort I’m putting into solving the problem.				−0.35		
9	I take additional action to try to get rid of the problem.				0.64		
16	I think about how I might best handle the problem.				0.69		
28	I think hard about what steps to take.				0.62		
31	I put aside other activities in order to concentrate on this.				0.4		
3R	I say to myself “this isn’t real.”					0.34	
10	I sleep more than usual.					0.55	
13R	I refuse to believe that it has happened.					0.64	
11	I daydream about things other than this					0.52	
22	I go to movies or watch TV, to think about it less.					0.37	
21	I have been making jokes about it.						0.68
32	I’ve been making fun of the situation.						0.83

Note. The results of the 6-factor solution are presented. The first column shows the item number in the questionnaire.

**Table 2:** Fit indices for six-factor model of COPE.

Fit Indice	CFI	TLI	RMSEA	SRMR
Value	0.988	0.986	0.057	0.064

**Table 3:** Final Factor Loadings (CFA).

No.	Item	Factor 1 Turning to Religion	Factor 2 Socio- Emotional Support	Factor 3 Acceptance	Factor 4 Problem- Focused Coping	Factor 5 Avoidance	Factor 6 Humor
4	I seek God’s help.	0.952					
8	I put my trust in God.	0.943					
25	I try to find comfort in my religion.	0.952					
29	I pray more than usual.	0.946					
6	I try to get emotional support from friends or relatives.		0.702				
12	I let my feelings out.		0.672				
14	I try to get advice from someone about what to do.		0.823				
17	I get sympathy and understanding from someone.		0.806				
24	I get upset and let my emotions out.		0.669				
26	I talk to someone who could do something concrete about the problem.		0.737				
19	I get used to the idea that it happened.			0.710			
23	I accept that this has happened and that it can’t be changed.			0.785			
27	I learn to live with it.			0.743			
30	I accept the reality of the fact that it happened.			0.878			
2	I concentrate my efforts on doing something about it.				0.584		
9	I take additional action to try to get rid of the problem.				0.709		
16	I think about how I might best handle the problem.				0.844		
28	I think hard about what steps to take.				0.690		
31	I put aside other activities in order to concentrate on this.				0.573		
10	I sleep more than usual.					0.593	
13	I refuse to believe that it has happened.					0.647	
11	I daydream about things other than this					0.673	
22	I go to movies or watch TV, to think about it less.					0.544	
21	I have been making jokes about it.						0.907
32	I’ve been making fun of the situation.						0.750
Internal consistency (Cronbach’s alpha)		0.91	0.82	0.80	0.76	0.65	0.73

Note. The results of the 6-factor solution are presented. The first column shows the item number in the questionnaire.

**Table 4:** Values of Pearson correlation coefficients between the scales of the Coping Strategies Questionnaire (Brief COPE) and other questionnaires.

	Brief COPE Turning to Religion	Brief COPE Socio-Emotional Support	Brief COPE Acceptance	Brief COPE Problem-Focused Coping	Brief COPE Avoidance	Brief COPE Humor
Brief COPE Turning to religion	1.00					
Brief COPE Socio-emotional support	**0.251** ***	1.00				
Brief COPE Acceptance	**0.055** ***	**0.381** ***	1.00			
Brief COPE Problem-focused coping	**0.165** ***	**0.453** ***	**0.506** ***	1.00		
Brief COPE Avoidance	**0.176** ***	**0.580** ***	**0.290** ***	**0.092** ***	1.00	
Brief COPE Humor	**0.067** ***	**0.185** ***	**0.384** ***	**0.373** ***	**0.192** ***	1.00
HADS Anxiety	**0.068** *	**0.254** ***	0.029	**−0.111** ***	**0.364** ***	−0.025
HADS Depression	0.01	−0.027	**−0.169** ***	**−0.29** ***	**0.181** ***	**−0.138** ***
MTQ	**0.092** **	**0.120** ***	**0.190** ***	**0.288** ***	0.016	**0.117** ***
PSS general score	−0.046	**0.235** ***	0.008	**−0.214** ***	**0.351** ***	**−0.067** *

Note. Significant correlations are highlighted in bold. * *p* < 0.05, ** *p* < 0.01, *** *p* < 0.001.

**Table 5:** Descriptive Statistics for the scales of the Short Version of the Coping Strategies Questionnaire (Brief COPE). N = 670.

Brief COPE Scales	Mean	Median	SD	Min/Max	Asymmetry	Excess
Avoidance	8.77	9.0	2.42	4/16	0.30	−0.25
Problem-focused coping	14.23	14.0	2.60	5/20	−0.27	0.20
Socio-emotional support	14.60	15.0	3.98	6/24	0.11	−0.44
Turning to religion	6.51	5.0	3.12	4/16	1.14	0.35
Humor	4.44	4.0	1.63	2/8	0.18	−0.77
Acceptance	11.15	12.0	2.38	4/16	−0.48	0.34

**Table 6:** Gender Differences and Descriptive Statistics for the Scales of the Short Version of the Coping Strategies Questionnaire (Brief COPE).

Brief COPE Scales	Mean, SD (Male)	Mean, SD (Female)	Mean Differences (M–F)	Student’s *t*-Test (*p*-Value)
Avoidance	8.35 (2.32)	8.88 (2.43)	−0.52	−2.30 (0.02) *
Problem-focused coping	14.34 (2.55)	14.22 (2.50)	0.11	0.47 (0.63)
Socio-emotional support	13.23 (3.72)	14.97 (3.98)	−1.74	−4.68 (<0.001) **
Turning to religion	5.75 (2.68)	6.73 (3.18)	−0.98	−3.31 (<0.001) **
Humor	4.77 (1.62)	4.35 (1.62)	0.43	2.78 (0.006) **
Acceptance	11.06 (2.46)	11.18 (2.37)	−0.13	−0.56 (0.57)

Note. N = 670; * *p* < 0.05; ** *p* < 0.01.

## Data Availability

The datasets generated and analyzed during the current study are available from the corresponding author upon reasonable request. None of the experiments were preregistered.

## Appendix A

**Table A1:** Theoretical structure of Brief COPE.

Initial Subscales Full Russian Version	Item in Brief Russian Version	Translation	Subscales of Brief Russian Version
Mental disengagement	1 Я погpyжaюcь в paботy или дpyгиe дeлa, чтобы отключитьcя от пpоблeм.	1 I turn to work or other substitute activities to take my mind off things.	Self-distraction
Mental disengagement	10 Я cплю большe обычного, cтapaяcь зaбыть о пpоблeмe.	10 I sleep more than usual.	
Mental disengagement	11 Я пpeдaюcь ϕaнтaзиям нa дpyгиe тeмы, чтобы отвлeчьcя.	11 I daydream about things other than this.	
Mental disengagement	22 Я читaю, cмотpю ϕильмы или тeлeвизоp или дeлaю что-то дpyгоe, чтобы отвлeчьcя.	22 I read, go to movies or watch TV, to think about it less.	
Active coping	2 Я cоcpeдоточивaю ycилия нa том, чтобы кaк-то peшить пpоблeмy.	2 I take direct action to get around the problem.	Active coping
Denial	3R Я говоpю ceбe: «этого нe можeт быть».	3R I say to myself “this isn’t real.”	
Behavioral disengagement	7R Я не предпринимaю aктивных действий.	7R I reduce the amount of effort I’m putting into solving the problem.	
Active coping	9 Я пpeдпpинимaю кaкиe-то eщe дeйcтвия, cтapaяcь пpeодолeть cложившyюcя cитyaцию.	9 I take additional action to try to get rid of the problem.	
Denial	13R Mнe нe xочeтcя вepить, что это пpоизошло.	13R I refuse to believe that it has happened.	
Planning	16 Я дyмaю, кaк лyчшe вceго я могy cпpaвитьcя c этой пpоблeмой.	16 I think about how I might best handle the problem.	
Behavioral disengagement	18R Я пepecтaю пытaтьcя добитьcя cвоeго (полyчить то, что я xочy).	18R I give up the attempt to get what I want.	
Planning	28 Я тщaтeльно обдyмывaю шaги, котоpыe бyдy пpeдпpинимaть для peшeния пpоблeмы.	28 I think hard about what steps to take.	
Suppression of competing activities	31 Я отклaдывaю дpyгиe дeлa в cтоpонy, чтобы cоcpeдоточитьcя нa peшeнии пpоблeмы.	31 I put aside other activities in order to concentrate on this.	
Turning to religion	4 Я пpошy помощи y Богa.	4 I seek God’s help.	Turning to religion
Turning to religion	8 Я нaдeюcь нa то, что Бог мнe поможeт.	8 I put my trust in God.	
Turning to religion	25 Я пытaюcь нaйти yтeшeниe в вepe (peлигии).	25 I try to find comfort in my religion.	
Turning to religion	29 Я молюcь (большe, чeм обычно).	29 I pray more than usual.	
Seeking of emotional social support	6 Я cтapaюcь полyчить эмоционaльнyю поддepжкy y дpyзeй или pодныx.	6 I try to get emotional support from friends or relatives.	Socio-emotional support
Focus on and venting of emotions	12 Я дaю выxод cвоим пepeживaниям.	12 I let my feelings out.	
Use of instrumental support	14 Я ищy cовeтa y дpyгиx людeй, что дeлaть дaльшe.	14 try to get advice from someone about what to do.	
Seeking of emotional social support	17 Я ищy cочyвcтвия и понимaния y дpyгиx людeй.	17 I get sympathy and understanding from someone.	
Focus on and venting of emotions	24 Я пepeживaю и aктивно пpоявляю cвои чyвcтвa.	24 I feel a lot of emotional distress and I find myself expressing those feelings a lot.	
Use of instrumental support	26 Я говоpю c кeм-нибyдь, кто мог бы конкpeтно помочь peшить мою пpоблeмy.	26 I talk to someone who could do something concrete about the problem.	
Acceptance	23 Я cтapaюcь пpинять cитyaцию, cжитьcя c нeй.	23 I accept that this has happened and that it can’t be changed.	Acceptance
Acceptance	27 Я yчycь жить c этим.	27 I learn to live with it.	
Acceptance	30 Я cтapaюcь пpинять то, что cлyчилоcь, пpивыкнyть к этомy.	30 I accept the reality of the fact that it happened.	
Acceptance	19 Я cтapaюcь пpивыкнyть к мыcли, что это cлyчилоcь, aдaптиpовaтьcя к cитyaции.	19 I get used to the idea that it happened and adapted to it.	
Positive reinterpretation and growth	15 Я пытaюcь поcмотpeть нa cитyaцию c болee позитивной cтоpоны, в ином cвeтe.	15 I try to see it in a different light, to make it seem more positive.	Positive coping
Positive reinterpretation and growth	20 Я ищy что-то xоpошee в том, что пpоизошло.	20 I look for something good in what is happening.	
Humor	21 Я пepeвожy cлyчившeecя в шyткy.	21 I have been making jokes about it.	
Humor	32 Я нaxожy в cлyчившeмcя зaбaвныe момeнты.	32 I’ve been making fun of the situation.	
Alcohol–drug disengagement	5 Я выпивaю или пpинимaю лeкapcтвa, чтобы помeньшe дyмaть о пpоблeмe.	5 I drink alcohol or take drugs, in order to think about it less.	Substance-use

Notes: Russian translation is taken from Rasskazova et al.’s COPE version.

**Table A2:** Brief COPE.

No.	Item	1	2	3	4
		Never or Almost Never	Rarely	From Time to Time	Very Frequently
1	I concentrate my efforts on doing something about it.				
2	I seek God’s help.				
3	I try to get emotional support from friends or relatives.				
4	I put my trust in God.				
5	I take additional action to try to get rid of the problem.				
6	I sleep more than usual.				
7R	I refuse to believe that it has happened.				
8	I let my feelings out.				
9	I daydream about things other than this				
10	I try to get advice from someone about what to do.				
11	I think about how I might best handle the problem.				
12	I get sympathy and understanding from someone.				
13	I get used to the idea that it happened.				
14	I have been making jokes about it.				
15	I go to movies or watch TV, to think about it less.				
16	I accept that this has happened and that it can’t be changed.				
17	I get upset and let my emotions out.				
18	I try to find comfort in my religion.				
19	I talk to someone who could do something concrete about the problem.				
20	I learn to live with it.				
21	I think hard about what steps to take.				
22	I pray more than usual.				
23	I accept the reality of the fact that it happened.				
24	I put aside other activities in order to concentrate on this.				
25	I’ve been making fun of the situation.				
